# Threatened Corals Provide Underexplored Microbial Habitats

**DOI:** 10.1371/journal.pone.0009554

**Published:** 2010-03-05

**Authors:** Shinichi Sunagawa, Cheryl M. Woodley, Mónica Medina

**Affiliations:** 1 School of Natural Sciences, University of California Merced, Merced, California, United States of America; 2 Hollings Marine Laboratory, Center for Coastal Environmental Health and Biomolecular Research, National Oceanic and Atmospheric Administration's National Ocean Service, Charleston, South Carolina, United States of America; University of California San Diego, United States of America

## Abstract

Contemporary in-depth sequencing of environmental samples has provided novel insights into microbial community structures, revealing that their diversity had been previously underestimated. Communities in marine environments are commonly composed of a few dominant taxa and a high number of taxonomically diverse, low-abundance organisms. However, studying the roles and genomic information of these “rare” organisms remains challenging, because little is known about their ecological niches and the environmental conditions to which they respond. Given the current threat to coral reef ecosystems, we investigated the potential of corals to provide highly specialized habitats for bacterial taxa including those that are rarely detected or absent in surrounding reef waters. The analysis of more than 350,000 small subunit ribosomal RNA (16S rRNA) sequence tags and almost 2,000 nearly full-length 16S rRNA gene sequences revealed that rare seawater biosphere members are highly abundant or even dominant in diverse Caribbean corals. Closely related corals (in the same genus/family) harbored similar bacterial communities. At higher taxonomic levels, however, the similarities of these communities did not correlate with the phylogenetic relationships among corals, opening novel questions about the evolutionary stability of coral-microbial associations. Large proportions of OTUs (28.7–49.1%) were unique to the coral species of origin. Analysis of the most dominant ribotypes suggests that many uncovered bacterial taxa exist in coral habitats and await future exploration. Our results indicate that coral species, and by extension other animal hosts, act as specialized habitats of otherwise rare microbes in marine ecosystems. Here, deep sequencing provided insights into coral microbiota at an unparalleled resolution and revealed that corals harbor many bacterial taxa previously not known. Given that two of the coral species investigated are listed as threatened under the U.S. Endangered Species Act, our results add an important microbial diversity-based perspective to the significance of conserving coral reefs.

## Introduction

Microorganisms dominate the oceans' total biomass [1], phylogenetic diversity and metabolic activity. Only recently have advances in sequencing technology allowed large-scale exploration of taxonomic diversity, population structure, functional potential, and geographic distribution of marine microbes [2], [3], [4]. One of the most remarkable findings has been that diverse microbial taxa exist at very low abundances while accounting for much of the total diversity in various marine environments [3]. This “rare biosphere” stands largely unexplored, but is inherently linked to several important questions [5]. For example, it is unknown whether low-abundance microbes are restricted to particular environments or universally dispersed [6]. In theory, rare organisms may become abundant, if not dominant, in response to environmental or habitat changes, i.e., when conditions shift to become more suitable for rapid growth. However, the exact nature of these changes is yet to be determined. Therefore, the identification of environmental conditions and/or ecological habitats specialized to support rare and underexplored bacteria are critical steps toward a better understanding of the evolutionary mechanisms and ecological forces that drive the biogeography, population structure, and temporal dynamics of the marine microbial biosphere.

Coral reefs are among the most biologically diverse ecosystems in the world, but they are facing an alarming risk of further and more rapid decline [7]. Corals are sessile keystone species in tropical reef environments exposed to tidal mixing and reef water flow (ensuring high dispersal of planktonic microorganisms); thus, they represent a well-suited study system to investigate their role as specialized microbial habitats. Microbial communities in corals have been studied with regard to coral health and disease [8], [9], antimicrobial properties of coral mucus [10], [11], and their potential role in recycling organic matter within reef ecosystems [12]. It has also been hypothesized that microbial partners play important roles in the response (and potentially the adaptation) of the coral holobiont, i.e., the host organism with all its associated microbes, to environmental changes [13].

Diversity surveys using nuclear small subunit ribosomal RNA (16S rRNA) gene clone library sequence data have provided evidence that communities of coral-associated bacteria appear to be host species-specific, and differ from those dominating the surrounding reef water [14], [15], [16]. One major drawback of these studies is that conventional sequencing methods are limited if the complexity and diversity of microbial community populations are to be captured beyond the most dominant taxa [17], [18], [19]. With the advent of second-generation sequencing technologies (e.g., pyrosequencing), it is now possible to detect rare taxa [3] that may serve as a reservoir of functional diversity and potentially becoming dominant when environmental conditions change [20].

In this study, over 350,000 hypervariable region 6 small subunit ribosomal RNA (V6) tag sequences and 1,960 nearly full-length 16S rRNA gene sequences were generated according to previously published protocols [18], [21]. We directly compared bacterial community members living in close association with seven Caribbean coral species and those inhabiting the surrounding seawater to test the potential of corals to provide habitats for marine bacteria. Bacterial communities were also clustered by similarity to determine whether they displayed correlations with the known phylogenetic relationships among the sampled corals. Our study included two Caribbean coral species that are listed as threatened under the U.S. Endangered Species Act to assess the magnitude of biodiversity loss if we fail to preserve imperiled coral species.

## Results and Discussion

### Corals Provide Habitats for Extremely Diverse Bacterial Communities Including Rare Seawater Biosphere Members

**Figure 1:**
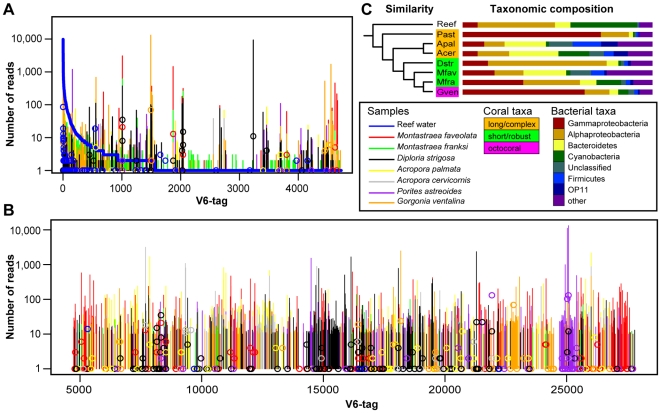
V6-tag abundance profiles, similarity clustering, and taxonomic composition of bacterial communities. (A) Rank abundance curve for V6-tags detected in reef water superimposed with abundances found in coral samples shown as vertical colored bars. (B) Abundances of V6-tags, which were detected exclusively in corals, are shown alphabetically sorted by taxonomic classification of V6-tags (x-axis). In (A) and (B), circles denote log-scaled abundances of nearly full-length 16S rRNA gene sequences that were mapped to the respective V6-tag sequences. (C) Taxonomic composition of all samples and dendrogram of OTU abundances showing similarities between samples, which are color-coded according coral host taxonomy. Details on the taxonomic composition of each sample can be found in Table S1. Abbreviations used: Acer = *Acropora cervicornis*; Apal = *Acropora palmata*; Dstr = *Diploria strigosa*; Gven = *Gorgonia ventalina*; Mfav = *Montastraea faveolata*; Mfra = *Montastraea franksi*; Past = *Porites astreoides*; Reef = reef water.

Ranking of the unique V6-tags sampled from reef water by abundance yielded a low number of dominant taxa, results similar to those previously reported for other marine environments [3], [5], [21]. More than 50% of all reads were found in 12 unique V6-tags and a large number of rare taxa accounted for most of the observed diversity (Figure 1A, Table S1). A superimposition of tags collected from the seven coral species revealed that many taxa rarely detected in the reef water were highly abundant, and in some cases represented the most dominant taxa in coral samples (Figure 1A). Furthermore, we identified more than 20,000 V6-tags that were unique to coral samples (Figure 1B). We corroborated the bacterial origin for many of these V6-tags by mapping the tag-sequences to nearly full-length 16S rRNA sequences that were generated from the same set of samples (Figure 1A and B). While we cannot discern whether our observations are a consequence of coral-associated bacteria becoming diluted in reef waters or are rare members of the water column finding suitable environments for rapid replication in coral hosts, these results demonstrate that corals provide specialized habitats for select groups of marine bacteria.

Estimates of species richness may be biased by errors in pyrosequencing data [22], [23] and uneven sampling efforts [24]. Thus, we first re-sampled all sequences to a common depth based on the dataset with the lowest number of reads (15,932 tags, Table S1), and second, clustered V6-tag sequences to the 97% similarity level (OTU_0.03_) before comparing the species richness in each coral sample (Table 1). Our results suggest that corals potentially harbor several thousand OTUs_0.03_, which is largely in accordance with previous diversity estimates [15], [18]. Corals that form massive, mound-shaped colonies (*Montastraea* spp., *Diploria strigosa*, and *Porites astreoides*) had higher estimated diversities than the branch-forming acroporid species (*Acropora* spp.) and the gorgonian coral *Gorgonia ventalina* (Table 1). Therefore, the possibility that morphology plays a role in determining the diversity of coral microbiota becomes an intriguing hypothesis. In total, more than 8,500 OTUs_0.03_ were detected and assigned to 31 phyla, with up to 26 phyla per coral species. This census of bacterial taxa in corals with 1,143–2,050 OTUs_0.03_ detected and 2,177–4,026 OTUs_0.03_ predicted (Table 1) suggests an extraordinary diversity of coral-associated bacteria, which is comparable to the one recently described for sponges [25].

**Table 1:** V6-tag sequencing statistics and species richness estimates.

Species/Sample	All tags	All unique tags	Re-sampled* unique tags	OTU_0.03_	Chao1_0.03_
*Montastraea faveolata*	46,350	5,764	2,681	1,553	2,925
*Montastraea franksi*	41,962	6,964	3,392	2,050	4,026
*Diploria strigosa*	40,073	4,908	2,618	1,759	3,801
*Acropora palmata*	60,390	3,790	2,431	1,671	2,576
*Acropora cervicornis*	37,095	2,489	2,476	1,616	2,602
*Porites astreoides*	44,004	4,464	2,048	1,340	3,106
*Gorgonia ventalina*	36,750	3,322	1,863	1,143	2,177
Reef water	44,190	4,735	2,036	1,079	1,996
total	350,814	27,854	15,023	8,515	14,243

*After removal of chloroplast-derived sequences, V6-reads were re-sampled based on the sample with the smallest number of reads (15,932).

Previous studies using clone library sequencing and community profiling data (T-RFLP and DGGE) suggest that bacterial communities in corals are distinct from those inhabiting the surrounding seawater [14], [16]. With virtually no overlap (e.g., only 5% of ribotypes were shared between seawater and dead coral surface libraries) between coral-associated and seawater bacteria [14], and vertical transmission of bacteria seemingly absent in at least one coral species [26], one important question arises: How (and when) do corals acquire the complex microbiota commonly found in adult colonies? Compared to previous studies, our massive sequencing approach has 1) increased the depth of sampling by more than two orders of magnitude, and 2) uncovered a vast number of bacterial taxa associated with an unparalleled number of coral species interrogated for their bacterial communities. More than 4,000 V6-tags were shared in coral and seawater libraries (Figure 1A), thus a lack of sequencing depth was a likely reason for the apparent absence of overlap between coral- and seawater-associated bacteria in previous studies. The detection of rare taxa in the water column, while being dominant in corals, has also important implications for the mode of bacterial symbiont acquisition in coral hosts. Given our results, it is reasonable to hypothesize that rare seawater biosphere members act as seed organisms for coral microbiota, implying that corals could acquire species-specific bacteria from the environment without any vertical mode of transmission. The possibility to capture the presence of rare taxa will thus be extremely valuable in studying both the onset of coral-microbial associations and the spatio-temporal variability in coral-associated microbiota.

### Similarities of Bacterial Communities Based on V6-Tag Abundance Profiles

Reef-building corals (Hexacorallia∶Scleractinia) are divided into two main phylogenic lineages (i.e., Short/Robust and Long/Complex clades), which are separated by approximately 240–288 million years of divergent evolution [27], [28]. We clustered V6-tag abundance profiles to ask whether a correlation exists between the bacterial community composition and the known coral host phylogeny (Figure 1C). Similarities between bacterial communities were found to partially reflect the known coral phylogeny, but also showed inconsistencies at higher taxonomic levels. For example, the most similar bacterial communities among all samples were derived from the congeneric species *Acropora palmata* and *A. cervicornis*. Furthermore, samples clustered by their clade affiliation, i.e., *Montastraea faveolata*, *M. franksi*, and *Diploria strigosa* (Short/Robust clade) were found in a different cluster than the more distantly related acroporid relatives (Long/Complex clade). Conversely, we found the octocoral-derived bacterial community to be inconsistent (i.e., clustering with Short/Robust clade communities) with the currently accepted phylogenetic placement of gorgonians in the anthozoan tree (i.e., outside Scleractinia). Likewise, *Porites astreoides* did not cluster with the acroporid species, although they belong to the same clade (Long/Complex).

Understanding of the acquisition, maintenance, and change of microbial communities in a coral host are fundamental questions in the study of coral-microbial associations. Vertical transmission of bacteria has, at least for one coral species, been excluded as a mode of symbiont acquisition [26], implying that corals may need to acquire their complex microbiota solely by environmental (horizontal) transmission throughout their lifespan. Thus, adult corals are likely to be associated with a mixed consortium of bacteria of which many may not co-evolve with their host. In fact, Littman et al. (2009) compared bacterial communities of three Indo-Pacific acroporid species at two environmentally distinct locations using DGGE, T-RFLP, and 16S RNA gene clone library sequencing, and found that samples grouped by location rather than coral species [29]. Thus, environmental factors appear to play an important role in driving coral-bacterial community composition, which is in accordance with temporal variations that had previously been reported for a Mediterranean coral species [30]. Our data did not allow for assessing inter-colonial variability, i.e., whether replicate samples from different corals would have clustered according to species. However, it is intriguing to note that an averaged, abundance-based index of community composition displayed similar profiles among closely related corals in the same genus (*Acropora* spp.) or family (*Montastraea* spp. and *D. strigosa*), but not at higher taxonomic levels. Thus, our results lend further support to the idea that coral-associated microbiota are similar among closely related coral species. Interestingly, coral-associated microbial communities were also found to show higher similarities in metabolic characteristics within groups of congeneric coral species (reviewed in [31]), while the topology of the clusters was found to be inconsistent with the higher clade designations of reef-building corals (e.g., the Short/Robust clade corals in the genus *Diploria* were grouped together with clusters of species belonging to the Long/Complex clade genera *Acropora* and *Porites*). Based on this information, corals may represent specialized habitats in which microorganisms have divergently evolved over many generations (e.g., due to the availability of nutrients specific to each coral host), but many coral-microbial associations may not be stable over long evolutionary time scales. More focused work is required in order to 1) better understand the ecological and evolutionary forces that drive coral-associated bacterial community composition, and 2) identify specific bacterial species that may be co-evolving with their hosts as has been exemplified in a number of animals including cnidarians [32], [33], [34], [35], [36].

### Threatened Coral Species Host Unique and Underexplored Bacterial Taxa

We examined whether different coral species provide distinct microbial habitats by testing the null-hypothesis that OTUs were randomly present or absent in any of the 8 samples. We binned unique OTUs_0.03_ into the 2^8^ ( = 256) possible permutations (e.g., V6-tags present in reef water only, tags present in reef water and *M. faveolata*, etc.) and found that among all possible combinations, those that represent the presence of OTUs_0.03_ in one particular environment only (e.g., present in reef water only, in *M. faveolata* only, etc.) ranked at the top 8 positions (Table 2). This result significantly deviates from a random distribution, and supports the hypothesis that coral species are likely to harbor a number of host-specific bacteria [15]. A search for the most closely related relatives to the most abundant coral-associated bacteria revealed that corals harbor many unclassified species and potentially novel genera or even higher taxonomic groups (Table 3). In the future, a sampling strategy that includes multiple individuals of the same coral species in different environments will help in 1) delineating host species-specific taxa from individual-to-individual variability, and 2) determining the role of environmental factors on the diversity and community structure of coral microbiota.

**Table 2:** Top 10 ranked OTU_0.03_ distribution possibilities.

OTUs_0.03_ present in	Number of OTUs_0.03_	Number (%) of all OTUs_0.03_ in sample/s
*Diploria strigosa* only	907	1,902 (47.7)
*Acorpora palmata* only	866	1,834 (47.2)
*Acropora cervicornis* only	768	1,793 (42.8)
*Montastraea faveolata* only	757	1,754 (43.2)
*Porites astreoides* only	701	1,429 (49.1)
*Montastraea franksi* only	636	2,448 (26.0)
Reef water only	601	1,183 (50.8)
*Gorgonia ventalina* only	383	1,333 (28.7)
*Montastraea franksi* and Reef water	202	3,193 (6.3)
*Montastraea franksi* and *Diploria strigosa*	180	3,772 (4.8)

**Table 3:** Best non-coral associated BLAST hits of most abundant OTUs isolated from corals.

OTU_0.03_ *	Accession	Best BLAST hit	% identity	Isolation source	Accession
Mfav_H04	GU118607	Endosymbiont of *Acanthamoeba* sp.	93	Protozoa	AF215634
Mfav_L18	GU118673	*Flexibacter aggregans*	94.2	Sand	AB078038
Mfra_I15	GU118732	Endosymbiont of *Acanthamoeba* sp.	93	Protozoa	AF215634
Mfra_G19	GU118699	Uncultured bacterium clone AG3	96.7	Fish	EU884929
Apal_J06	GU118139	*Desulforhopalus singaporensis*	97.3	Marine mud	AF118453
Apal_K21	GU118042	Uncultured bacterium clone 655952	87.6	Sediment	DQ404824
Acer_F19	GU117998	*Leptolyngbya* sp. 0BB32S02	94.6	Freshwater	AJ639894
Acer_F06	GU117952	*Flexibacter aggregans*	94.3	Sand	AB078038
Past_G20	GU119165	Uncultured bacterium clone AG3	96.7	Fish	EU884929
Past_A20	GU118916	Uncultured *Desulfocapsa* sp. clone CBII115	95.5	Marine sediment	DQ831556
Gven_C04	GU118411	Spongiobacter nickelotolerans	93.6	Marine sponge	AB205011
Gven_C22	GU118496	*Aquaspirillum peregrinum* subsp. *integrum*	86.5	Shellfish	EF612768
Dstr_B21	GU118204	Uncultured bacterium clone P9X2b3F06	94.2	Seafloor lava	EU491139
Dstr_G05	GU118184	Uncultured alpha proteobacterium HOC19	99.5	Marine sponge	AB054153

*selected cluster representative.

Host-associated bacterial communities can be shaped by a number of biological and physicochemical factors. For example, in humans, inter-microbial competition, pH and the activity of the immune system are all known to influence the formation of the microbiota [37], [38]. Likewise in corals, antimicrobial activity has been detected and suggested to play a role in the regulation of microbial populations [10], [11]. In addition, it is important to notice that reef-building corals live in symbiosis with photosynthetic dinoflagellates, which are responsible for strong diurnal fluctuations in pH and O_2_ concentrations within coral tissues [39], [40]. These parameters change along a steep gradient ranging from the coral tissue layer across a diffusive boundary layer that separates the coral tissue microenvironment from the surrounding seawater [39], [40]. We suggest that a combination of coral host immunity, inter-microbial interactions, physicochemical parameters (e.g., nutrients, pH, O_2_), and other factors, are likely to create adaptive landscapes that explain the ecological niche partitioning observed in this study. Furthermore, the adaptation to physico-chemically distinct microenvironments in different coral hosts over evolutionary time scales has potentially generated an untapped source of genomic innovation.

### Conclusions

At the beginning of the 21^st^ century, we find ourselves in an era where second-generation sequencing technologies have exposed a vast amount of undiscovered microbial diversity governing the Earth's biosphere. The high rate of discoveries ironically parallels the worldwide decline of many ecosystems, including coral reefs. Often considered as the proverbial canary in the mine for the oceans, their survival is currently at stake [7], while the ecological role and genomic makeup of the coral microbiome still remain largely unknown. Here, the application of 16S pyrotag sequencing revealed an extraordinary bacterial diversity in reef-building coral species, and provided first insights into the link between coral host communities and rare seawater biosphere members. Inconsistencies in the correlation between bacterial community composition and coral host phylogenies emphasized the need to better understand the ecological and evolutionary forces that determine the structure and composition of coral microbiota. The application of massively-parallel tag sequencing, along with hybridization-based methods [18] and metagenomics studies [9], [41], [42], represents a promising tool to complement established methods in rapidly advancing our knowledge about the ecological role, population structure, and dynamics of microbial communities in healthy vs. stressed/diseased states of corals [43].

Our study included two coral species, *Acropora palmata* and *A. cervicornis*, that once represented the most abundant species in Caribbean coral reefs, but are now listed as threatened under the U. S. Endangered Species Act. Given that our results are based on only a handful of coral species, it is reasonable to predict that future sequencing of coral- and other host-associated microbiota is likely to uncover a wealth of yet unknown genetic and functional diversity. Thus, our findings substantiate the justification [44] and highlight the importance of conserving coral reefs as an underexplored ecosystem from a microbial diversity-based perspective.

## Materials and Methods

### Ethics Statement

Experimental research followed internationally recognized guidelines according to CITES (the Convention on International Trade in Endangered Species of Wild Fauna and Flora) permit numbers: SEX/A-26-08 and SE/A-13-08. No ethical approval was required for any of the experimental research described here.

### Sample Collection and Isolation of Nucleic Acids

On the 26th and 27th of March 2008, replicate samples (N = 5) from seven Caribbean coral species (depth range: 1.5–5.5 m) were collected together with a reef water sample at “Crawl Cay” reef near Bocas del Toro in Panamá (9°15′N; 82°07′W) by SCUBA using a hammer and chisel. All collected coral fragments (size: 1–4 cm^2^) were placed in plastic bags, rinsed with 0.22 µm filtered seawater, and flash frozen in a dry shipper within less than one hour after collection. The reference water sample was collected (depth: ∼1 m) at the Crawl Cay buoy using a 5 L plastic bottle and filtered using a 0.22 µm Sterivex™ filter unit (Millipore), which was immediately flash frozen after filtration. At UC Merced, the coral fragments were homogenized on dry ice using a mortar and pestle and the frozen filter cut into smaller pieces (∼25 mm^2^). Approximately 50 mg of the resulting coral powder or the frozen filter pieces were used to extract DNA using the PowerPlant DNA extraction kit (MoBio Laboratories, Carlsbad, CA, USA). To increase the yield and quality of DNA preparations, we modified the manufacturer's instructions by: 1) adding 0.19 µL lysozyme (Epicentre; final: 10 U µL^−1^) to the Bead Solution/sample mixture, followed by an incubation of 10 min at room temperature, 2) adding 25 µL proteinase K (Invitrogen; final: ∼0.8 mg mL^−1^) to the lysozyme-treated mixture, followed by an incubation for 60 min at 65°C, and 3) adding 400 mg of each 0.1 and 0.5 mm zirconia/silica beads before samples were homogenized for 30 s using a Mini-BeadBeater-8 (Biospec Products, Inc., Bartlesville, OK, USA) instead of a Vortex Adapter (MoBio).

### V6-Tag and Nearly Full-Length Sequence Generation

The amplification of V6-tag amplicons from total DNA of coral samples required optimizations of previously published methods [3], [21]. After pooling replicate DNA extractions of each sample/species, we used Platinum High-Fidelity Taq Polymerase (Invitrogen) in combination with Buffer G (Epicentre), 50 ng of template DNA and keyed primers [21] in triplicate 30 µL PCR reactions and ran the following cycling conditions: [94°C–2′, (94°C–30″, 56°C–30″, 68°C–30″)×30, 68°C–2′]. The triplicate reactions were pooled and amplicons purified (Qiagen), before they were run out on a 2% agarose gel, excised (Qiagen), and purified to remove non-specific amplification products. Samples were sent to the Marine Biological Laboratory in Woods Hole, MA, for pyrosequencing. The resulting data were processed in February 2009 according to previously published methods [3], [21]. V6-tag sequences have been deposited in the National Center for Biotechnology Information (NCBI) Short Read Archive (SRA) under the project number SRP001172 and are publicly available from the Visualization and Analysis of Microbial Population Structure (VAMPS) database at http://vamps.mbl.edu.

In addition to V6-tag amplicons, nearly full-length 16S rRNA gene amplicons were generated from each sample and clone libraries generated following standard methods [18]. Transformed clone library stocks were sequenced by Agencourt Bioscience.

### Sequence Data Processing, Clustering, and Diversity Estimates

Chromatograms of nearly full-length 16S rRNA gene sequences were assembled, trimmed, quality-checked, and chimeric sequences removed as previously described [18]. The resulting sequences were classified using two different software pipelines (STAP [45] and RDP [46]), which were identical in their outcome regarding the identification of chloroplast-derived sequences (data not shown). All nearly full-length 16S rRNA gene sequences were deposited to the DDBJ/EMBL/GenBank databases under accession numbers: GU117926-GU119887.

Unique V6-tag sequences and their abundance data for each sample were downloaded from the VAMPS database. A local MySQL database was updated, and queried at different stages of the analysis to obtain general statistics or data matrices for further analyses. After downloading the processed sequence data, misclassified (as Firmicutes) chloroplast-derived V6-tags were identified by mapping all tags to nearly full-length sequences and subsequently removed. The rank abundance curve for tags detected in seawater was plotted and superimposed with tags present in coral samples using functions in R. V6-tags that were detected in clone library-derived 16S rRNA gene sequences were identified as described above and their quantities visualized in Figure 1A and B.

In order to account for uneven sampling efforts, V6-tags were re-sampled to a common total of 15,932, i.e., the number of unique sequences in the sample with the lowest number of sampled tags, using Daisy_chopper (v1.0) [24]. Re-sampled sequences were aligned and a pair-wise distance matrix calculated with the software programs MUSCLE [47] and quickdist [3], [48]. V6-tags were clustered into operational taxonomic units at the 97% similarity level (OTU_0.03_) and Chao1 nonparametric richness estimates calculated using the program DOTUR [49].

### Sample Clustering and Distribution of OTUs_0.03_

Determining the similarities of bacterial communities based on V6-tags using phylogenetic methods [50] was not feasible since many tags had no significant match to any sequence in the complete Greengenes [51] (45%; release November 2008) or Silva [52] databases (31%; release 100). Thus, we clustered V6-tags based on OTU_0.03_ data using Bray-Curtis distances and inferred a dendrogram with the unweighted pair-group average algorithm (UPGMA) as implemented in the NEIGHBOR program of the PHYLIP package [53]. The taxonomic composition of samples are based on the V6-tag classifications provided by the VAMPS database and were visualized using iTOL [54]. For the distribution of OTUs_0.03_ among all samples, we assigned one of 256 ( = 2^8^) possible combinations of presence or absence to each OTU_0.03_, and ranked the combinations by OTU abundance.

### Similarity Searches of Most Abundant 16S rRNA Sequences

For similarity searches of 16S rRNA gene sequences, all sequences within each sample were aligned using NAST [55], a distance matrix calculated using the DNADIST tool of the PHYLIP package through the Greengenes website [51], and clustered to the 97% similarity level as described above. The best cluster representative was identified based on sequence length and average quality values. A BLASTn (megablast) search was performed against the GenBank nucleotide database to identify the most similar non-coral-derived sequence entry.

### Bioinformatics

Data were analyzed using MySQL database queries, custom and published [24] Perl scripts, and UNIX commands (custom commands and scripts available upon request). Statistical analyses were performed in the R software environment [56] and visualization of data was supported by R scripts and iTOL [54].

## Supporting Information

Table S1 Re-sampled V6-tag abundances in coral samples and reef water.
(1.95 MB XLS)
